# pH-Taxis of Biohybrid Microsystems

**DOI:** 10.1038/srep11403

**Published:** 2015-06-15

**Authors:** Jiang Zhuang, Rika Wright Carlsen, Metin Sitti

**Affiliations:** 1 Department of Mechanical Engineering, Carnegie Mellon University, Pittsburgh, PA 15213, USA; 2 Department of Engineering, Robert Morris University, Pittsburgh, PA 15108, USA; 3 Max Planck Institute for Intelligent Systems, Stuttgart 70569, Germany

## Abstract

The last decade has seen an increasing number of studies developing bacteria and other cell-integrated biohybrid microsystems. However, the highly stochastic motion of these microsystems severely limits their potential use. Here, we present a method that exploits the pH sensing of flagellated bacteria to realize robust drift control of multi-bacteria propelled microrobots. Under three specifically configured pH gradients, we demonstrate that the microrobots exhibit both unidirectional and bidirectional pH-tactic behaviors, which are also observed in free-swimming bacteria. From trajectory analysis, we find that the swimming direction and speed biases are two major factors that contribute to their tactic drift motion. The motion analysis of microrobots also sheds light on the propulsion dynamics of the flagellated bacteria as bioactuators. It is expected that similar driving mechanisms are shared among pH-taxis, chemotaxis, and thermotaxis. By identifying the mechanism that drives the tactic behavior of bacteria-propelled microsystems, this study opens up an avenue towards improving the control of biohybrid microsystems. Furthermore, assuming that it is possible to tune the preferred pH of bioactuators by genetic engineering, these biohybrid microsystems could potentially be applied to sense the pH gradient induced by cancerous cells in stagnant fluids inside human body and realize targeted drug delivery.

Biohybrid microsystems, which integrate motile microoganisms or cells with engineered functional synthetic materials, have been heavily studied recently because of their potential applications in medicine, bioengineering, and environmental monitoring^1^. There is a particular interest in employing flagellated bacteria as onboard actuators and sensors in biohybrid systems due to their high motility, strong viability, versatile sensing abilities, and ease of genetic modification^2^,^3^,^4^,^5^,^6^,^7^,^8^,^9^,^10^,^11^,^12^. To utilize the swimming locomotion of these systems in applications such as targeted drug delivery and therapeutics, it is necessary to develop and implement reliable control methods. Using various physical steering methods, the control of several types of microrobotic systems at the single agent level has been demonstrated^13^,^14^,^15^. However, control of these microsystems at the swarm level has been challenging, partly because of the high inherent stochasticity of such systems. The large variability in the motion of biohybrid microsystems results from factors such as the randomness in the assembly process and the inherently stochastic and heterogeneous behavior of the assembled bioactuators. Some methods have been developed to improve the control of these microsystems. Koumakis *et al*. devised an anisotropic substrate to realize targeted delivery of bacteria-propelled colloids^16^, but the method required the use of sophisticated substrate patterns, which largely limits its applicability. Varying degrees of tactic behavior has also been reported in studies that rely on bacterial chemotaxis as a means to control bacteria-propelled microsystems^17^,^18^,^19^,^20^. However, the tactic motion of the microrobotic systems has yet to be extensively quantified, and the driving mechanism behind the behavior remains unclear to date.

Maintaining an appropriate pH level is vital to the survival of most microoganisms like bacteria, and they have evolved various sensing and regulatory strategies to adjust their cytoplasmic pH^21^,^22^. Flagellated bacteria such as *E. coli* have also been found to exhibit bidirectional pH-tactic behavior^23^,^24^,^25^, i.e., movement away from both strong acidic and alkaline pH environments. Given the pH tactic response of these bacterial strains and knowing that cancerous tumors have a lower pH compared to that of periphery normal tissue^26^,^27^, it would be enticing to explore the potential of applying pH-taxis based control of microrobotic systems for targeted drug delivery applications. To further explore the feasibility of such an approach, greater insight into the pH-tactic behavior of these microsystems is required.

Here, we present a method that takes advantage of the bacterial sensing of ambient pH to realize robust drift control of multi-bacteria propelled microsystems. *S. marcescens* is employed as the bioactuator for the microsystem, not only because it is a typical flagellated bacterial strain with high motility and tactic behaviors, but also because of its natural adhesion to negatively-charged, hydrophobic surfaces^28^,^29^, which greatly simplifies the assembly process of the microsystem. *S. marcescens* bacteria swim in liquid environments by incessant alternation of run and tumble states similar to *E. coli*^30^, with an average tumble rate measured to be around 1.3 s^−1^^29^. Their mean swimming speed can be as high as 47 *μ*m/s^31^. Temperature responses and chemotaxis of *S. marcescens* have been characterized and have also been found to resemble those of *E. coli*^29^,^32^. Since a common signaling machinery is suggested for chemotaxis, thermotaxis, and pH-taxis^25^, it is expected that *S. marcescens* also possesses a similar pH-tactic behavior as *E. coli*.

To perform a drift control study of the microsystems, we use a microfluidic device to generate three stable pH gradients. The bidirectional pH-taxis of free swimming bacteria is observed for the first time using the configured pH gradients; tracking of the swimming bacteria allows us to determine that the bacterial pH-tactic motion is mediated by the biased flagellar tumble rates. Then we study the distribution and motion of *S. marcescens* propelled microrobotic swarms. Depending on the applied pH gradient profile, the microrobotic systems are shown to exhibit either bidirectional or unidirectional tactic motions. Since it is not intuitively clear how a microrobot with multiple bacteria attached in random directions can produce the same pH-tactic response as free-swimming bacteria, we perform a detailed analysis on the trajectories of the microrobots, which enable us to determine that two motion bias factors contribute to the tactic drift velocity of the microrobotic systems.

## Results

### pH gradient generation

Using a diffusion based microfluidic gradient generator, three stable pH gradient profiles (named Gradient 1, 2 and 3 for convenience) were created in a quiescent fluidic channel, where samples of bacteria and bacteria-propelled microrobots were loaded and tested. Gradient 1 was created to study the bidirectional pH-taxis of bacteria and to demonstrate that the bacteria-propelled microrobots can be navigated by the ambient pH distribution, while Gradients 2 and 3 were used to quantify the unidirectional drift of the microrobots and therefore unveil the multi-bacterial driving and steering mechanism. Details about the gradient generator and visualizations of the pH gradients are included in the methods section.

### Bacterial bidirectional pH-taxis

**Figure 1:**
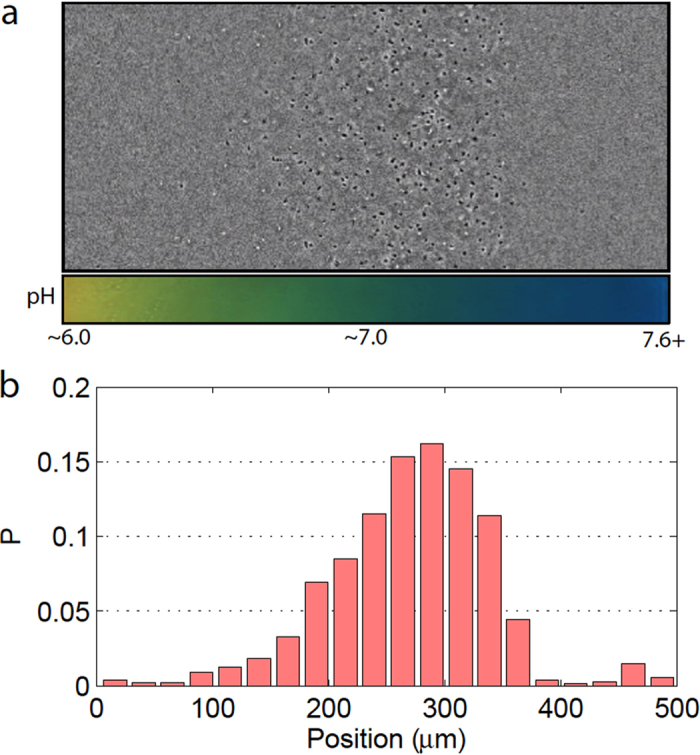
Bacterial bidirectional pH-taxis. (**a**) A phase contrast image of the free swimming *S. marcescens* at steady state (stabilized after around 1.5 min) under Gradient 1, where the black and white dots are cell bodies of bacteria. (**b**) Probability distribution of bacteria position extracted from multiple images at steady state.

Based on recent fluorescence resonance energy transfer (FRET) results as well as mathematical models, it has been proposed that *E. coli* is capable of taxis away from both strongly acidic and alkaline environmental conditions, resulting in accumulation of the bacteria at an optimal pH region^23^,^24^. However, such bidirectional taxis has never been visualized directly, and the associated swimming behavior has not been studied. Using Gradient 1 (pH: 6.0-7.6), which is a stable pH gradient that covers the pH transition from acidic to alkaline, we were able to observe the bidirectional pH-taxis of *S. marcescens* directly. As shown in Fig. 1(a,b), the bacteria accumulate to form a band around the center line of the sample channel after about 1.5 min from the start of the experiment; the position of the band corresponds to a pH value slightly above 7.0. From the color chart of the pH indicator, the optimal pH was found to correspond to values between 7.0 and 7.3. The distribution profile shows a sharper decrease in bacterial number in the transition from ambient to alkaline pH than the transition from ambient to acidic pH; this is probably due to the drastic pH change at this corresponding location.

Bacteria in an isotropic environment follow a purely random walk, which generates a uniform distribution of bacteria in a bounded space at steady state; a nonuniform distribution of incessantly moving bacteria in our sample channel reveals a motion deviating from a random walk. A biased distribution of bacteria is often seen in bacterial chemotaxis, which has been attributed to a biased tumble rate distribution based on swimming direction. Since FRET results^23^ indicate that similar signaling pathways are employed in both bacterial pH-taxis and chemotaxis, it is reasonable to expect that the banded distribution of *S. marcescens* under a pH gradient is also a result of a biased tumble rate. By tracking the bacterial swimming direction and detecting the number of tumble events in two rectangular regions with pH values below and above the optimal value, we find that the tumble rate distribution is significantly biased and is dependent on the swimming direction (Fig. S1). For both regions, the average tumble rate (based on ~2000 trajectories) of the bacteria is found to be substantially lower when swimming toward the optimal pH (~1.0 s^−1^) than when swimming toward the opposite direction (~1.5 s^−1^). Our results corroborate the reported bidirectional pH-taxis signaling pathway model^24^ and indicate a resemblance between *E. coli* and *S. marcescens* in terms of pH-tactic behavior.

### Bacteria-propelled microrobotic system controlled by pH gradient

**Figure 2:**
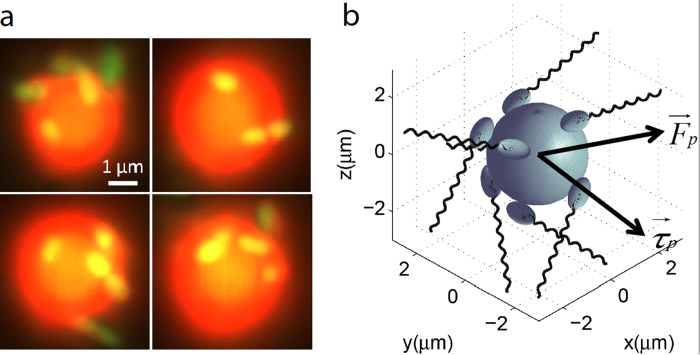
Bacteria-propelled biohybrid microrobotic system. (**a**) Sample fluorescent images of the microrobots, which are composed of multiple attached bacteria (yellow-green) and a spherical polystyrene bead (red). (**b**) At each moment, the applied forces on the bead from the attached bacteria can be represented by a net force ***F***_***p***_ and a net torque ***τ***_***p***_ vector.

We fabricated the biohybrid robots by randomly attaching multiple bacteria onto 3 *μ*m diameter polystyrene beads. Fluorescent staining enabled the simultaneous visualization of the attached bacteria and bead (Fig. 2 (a)). The mean and standard deviation of the bacterial attachment number were determined to be 9.0 ± 3.4, based on examinations of 20 microrobots randomly picked from the whole population. The position and orientation of the attached bacteria varied significantly from bead to bead, which is an expected result based on the spontaneous attachment of bacteria during the assembly process. Fig. 2(b) shows a simplified depiction of bacteria attached to a bead; the force vectors represent the instantaneous net force and torque exerted on the bead by the attached bacteria.

**Figure 3:**
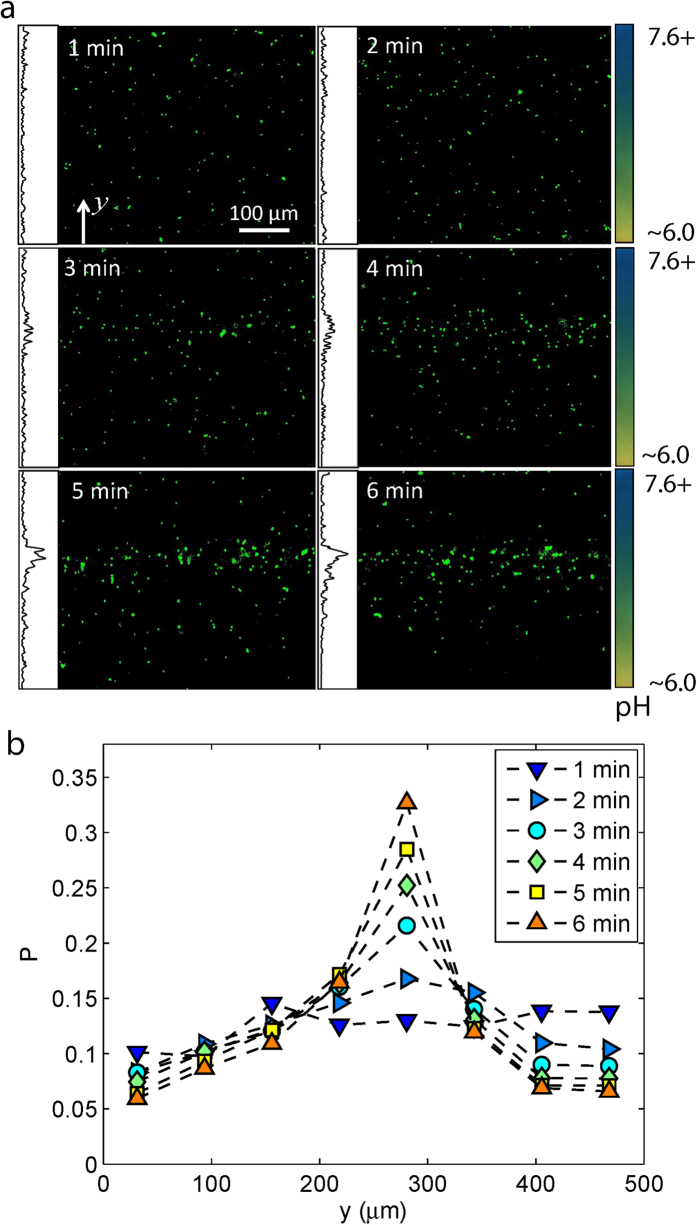
Bidirectional drift control of the microrobotic system. (**a**) Fluorescent images of the microrobots (green dots) show the evolution of the microrobot distribution in a fixed focal plane over time. The height (*y*-dimension) of each image covers the full channel width. The inset on the left side of each panel indicates the intensity profile of the frame along the *y*-axis. The color bars on the right hand side indicate the ambient pH gradient profile (Gradient 1). (**b**) Probability distribution of the microrobot position across the width of the sample channel, where each distribution is averaged from 200 fluorescent images taken around the corresponding time point.

By exposing a large number of microrobots to Gradient 1, we demonstrate drift control of the microrobots. As shown in Fig. 3(a), the microrobots were initially uniformly distributed. Over time, the uniform distribution evolves into a dense band of microrobots located around the centerline of the channel. This steady state distribution is achieved after about 6 minutes. The most probable location of the microrobots at steady state, which is shown in Fig. 3(b), coincides with that of free swimming bacteria (the optimal pH value). A side by side comparison between the distribution profile of free swimming bacteria and microrobots indicates a high degree of resemblance; in both distributions, there is a sharp decrease on the alkaline side of the optimal pH.

**Figure 4:**
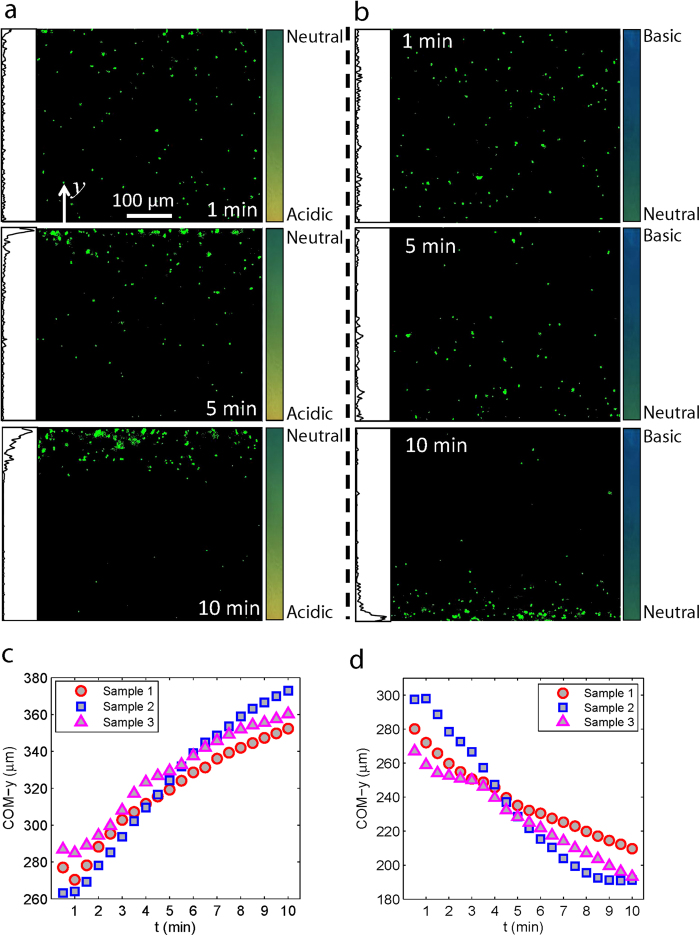
Unidirectional drift control of the microrobotic system. Fluorescent images show the migration of the microrobotic system away from more acidic (**a**) and more alkaline (**b**) conditions in the sample channels, where Gradients 2 and 3 were applied, respectively. The inset on the left side of each panel in (**a**) and (**b**) indicates the intensity profile of the frame along the *y*-axis. Plots of the variations of the COM-*y* of the microrobotic system over time: away from more acidic (**c**) and away from more alkaline regions (**d**). The results for three different system samples are shown for each case.

While the bidirectional pH-taxis shows the versatility of bacterial pH-taxis, it is preferable to study the bias factors that drive the tactic behavior under unidirectional taxis. Therefore, two more pH gradients, Gradient 2 (pH: 3.8-5.4) and Gradient 3 (pH: 8.2−9.8) (see more details in the methods section), were created to achieve the unidirectional drift control of the microrobotic system. It took approximately 10 minutes for the majority of microrobots to accumulate on one side of the channel in the two cases studied: taxis away from a more acidic condition (Fig. 4(a)) and taxis away from a more alkaline condition (Fig. 4(b)). Using image processing, the *y* component of the center of mass (COM-*y*, see details in methods section) of the microrobotic system can be computed at different time points. In Fig. 4(c,d), the drift behavior is shown to be highly consistent among the three tested samples for the two cases. Because the microrobots eventually accumulate at the wall of the device, they effectively don’t drift anymore but are still taken into account when calculating the average COM-*y*. This results in an artificial decrease in the slope of the COM-*y* over time as shown in Fig.4. Thus, the time derivative of the COM-*y* produces an underestimation of the actual pH-taxis drift velocity of the microrobotic system; a more accurate characterization of the pH-taxis drift velocity is obtained in the following section via analysis of the swimming trajectories of the microrobots.

### Trajectory analysis

To understand how a microrobot, which consists of a microsphere propelled by a group of randomly oriented bacteria, is endowed with pH-taxis capabilities, we tracked individual microrobots subjected to unidirectional pH-taxis. Motions of slowly moving microrobots are susceptible to ambient flows (0.41 ± 0.21 *μ*m/s); thus we only included the trajectories of microrobots with mean speed greater than 4 *μ*m/s (~10 times the ambient flow speed) in our analysis. In addition, trajectories that came within 50 *μ*m (~15 times the body length of the microrobot) of the walls were removed from the analysis since these microrobots were subjected to wall effects. Using these two criteria, over 900 trajectories (see Fig. S2 for sample trajectories) were collected and analyzed for each swarm sample undergoing unidirectional taxis.

### Heading distribution

**Figure 5:**
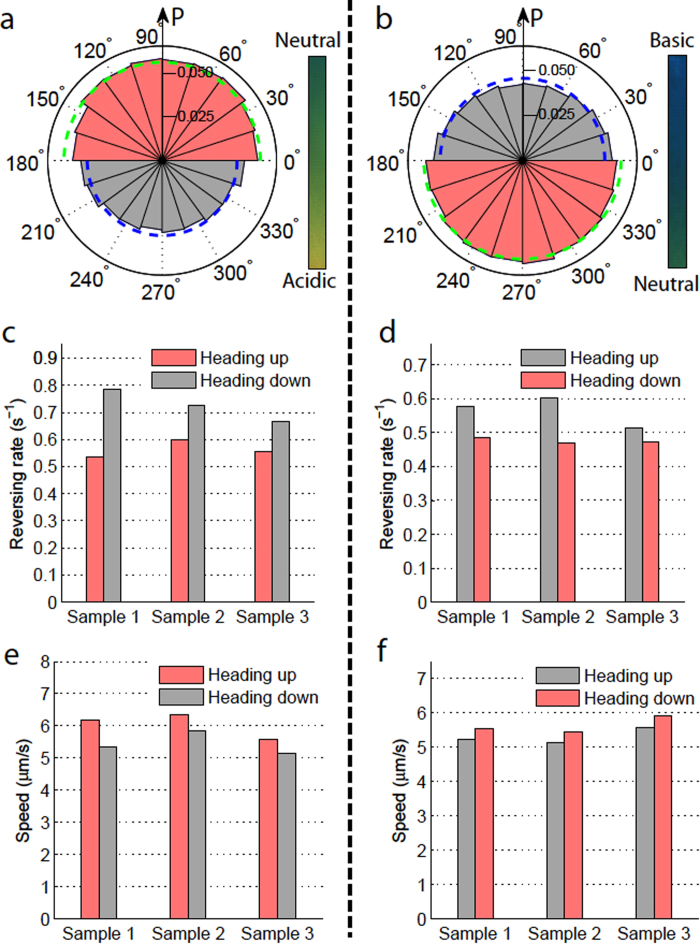
Biases in swimming heading, reversing rate (along *y*-axis), and 2D average swimming speed under two unidirectional pH-taxis cases: away from a more acidic condition (**a**,**c**,**e**) and away from a more alkaline condition (**b**,**d**,**f**). (**a**,**b**) Probability distributions of the swimming heading in 2D. (**c**,**d**) Heading direction reversing rate with respect to the heading direction along the *y*-axis. (**e**,**f**) Mean 2D swimming speed with respect to the heading direction along the *y*-axis. The pink color indicates results from motion towards favored pH regions while the gray color shows the corresponding results from motion towards unfavored pH regions.

The heading direction of all the trajectories (from three swarm samples) were determined on a frame-by-frame basis. By counting the number of frames where the swimming direction (i.e. orientation angle) was within a defined angle interval, the probability distributions of the swimming heading could be generated. The distributions also represent the portion of time that the microrobots spend swimming in each direction. Substantial biases in heading distributions are observed in Fig. 5(a,b). The least probable swimming directions correspond with the unfavorable conditions and the most probable directions correspond with the favorable conditions. The heading distribution provides a quantification of the swimming angle preference of the microrobots; however, it is not intuitively clear how the heading bias is achieved. Our following study on the swimming direction reversing rate along the *y*-axis (pH gradient direction) sheds light on the answer.

### Direction reversing rate

In Fig. 5(c–f), we study the motion bias of the microrobots with respect to their heading directions. Since the *y* component of the swimming velocity determines whether the microrobot is swimming towards the optimal pH region or not, we classified the headings of all frames into two groups in terms of their *y* direction: heading towards the optimal pH (shaded in red) and heading away from the optimal pH (shaded in gray). The direction reversing rate of each heading group is simply defined as the total number of *y*-direction switchings (from +*y* to −*y* or vice versa) observed in that group divided by the total number of frames of that heading group. As shown in Fig. 5(c,d), across three different samples, the direction reversing rates for microrobots moving towards favored pH regions are consistently smaller than for those moving towards unfavored pH regions. In other words, the orientation of the bacteria propelled microrobots is more persistent and less likely to change when they move towards the favored pH regions; this yields a larger portion of time spent moving towards an optimal pH region, as revealed by the heading bias in Fig. 5(a,b).

### Swimming speed

In addition to the heading bias, there is also a bias in the mean swimming speed with respect to the swimming direction (Fig. 5(e,f)); namely, a higher speed is observed when moving toward the favored pH region. Due to the low Reynolds number of the swimming motion, the hydrodynamics of the microrobot can be described by Stokes law, *F*_*d*_ = 6*πηRv*, where *F*_*d*_ is the fluidic drag force, *η* is the dynamic viscosity of the fluid medium, *R* is the radius of the spherical bead, and *v* is the instantaneous translational speed of the microrobot. The fluidic drag force *F*_*d*_ is balanced by the instantaneous resultant propulsive force (*F*_*p*_ in Fig. 2) produced by the attached bacteria. From the linear relationship between *F*_*p*_ and *v*, we can conclude that on average the attached bacteria exert a higher propulsive force on the microrobot when it move towards a favored pH environment.

### Drift velocity

In the chemotaxis or pH-taxis of free swimming bacteria, the heading bias is the only factor that contributes to the tactic drift. However, in microrobotic systems, both a swimming speed bias and a heading bias contribute to the drift velocity. To evaluate the dependency of drift velocity on these two factors, we derive a drift velocity equation based on a one-dimensional (1D) biased random walk scenario (see methods section for derivation),


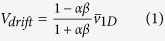


where *α* and *β* are the coefficients of the swimming speed bias and heading time bias, respectively, and 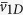 is the mean 1D swimming speed. The drift velocity of the microrobots in the gradient direction can be readily computed from our measurements on the heading bias, speed bias, and the mean swimming speed along the pH gradient direction. The average headings (the dashed arcs in Fig. 5) are used to evaluate the average heading time bias *β* (see the methods section). The drift velocities of the two cases (moving away from more acidic pH regions and moving away from more alkaline pH regions) were calculated to be similar, both of which are around 0.5 *μ*m/s. We further examined the relative contributions of the heading bias and speed bias to the overall drift velocity: the heading bias contributes to ~75% of the total drift velocity while the speed bias contributes to ~25% of the total. This indicates that, in addition to the heading bias, the speed bias is an essential mechanism of the microrobot’s tactic motion; this is a departure from the mechanisms known to cause the biased random walk observed in free swimming bacteria under pH-taxis or chemotaxis.

### Dependence on swimming speed

**Figure 6:**
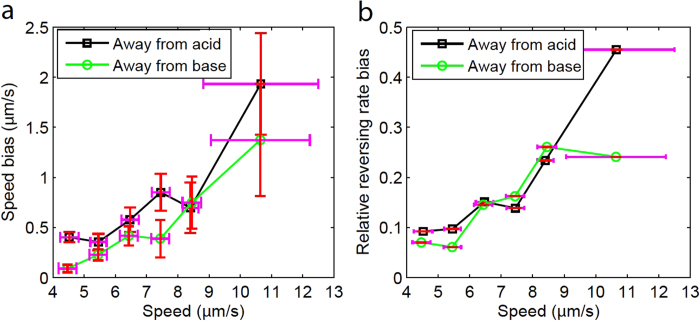
Dependences of motion biases on the average swimming speed. (**a**) Swimming speed bias with respect to the mean swimming speed. (**b**) Relative reversing rate bias with respect to the mean swimming speed. The horizontal error bars indicate the standard deviation of mean speeds for the trajectories grouped within a given speed interval (4–5, 5–6, 6–7, 7–8, 8–9, and ≥9 *μ*m/s). The vertical error bars in (**a**) denote the standard deviation of the speed bias for the trajectories falling within the corresponding speed intervals. In both (**a**) and (**b**), each speed interval has over 80 sample trajectories measured.

Since a wide variance in the swimming speeds of the microrobots was observed, it is meaningful to inquire about the potential influence of the absolute swimming speed on the motion bias. To analyze the dependence on swimming speed, the captured trajectories for each unidirectional pH-taxis case were divided into groups based on their mean instantaneous speed, as shown along the *x*-axis in Fig. 6. The relative reversing rate bias ((*r*^−^ − *r*^+^)/(*r*^−^ + *r*^+^), where *r*^+^ and *r*^-^are reversing rates towards and away from optimal pH, respectively) quantifies the dependence of the drift velocity on the direction reversing rate^33^. Both speed and relative reversing rate biases increase with increasing mean swimming speed, and this trend holds true for both cases (away from acid, away from base). Since the speed bias and reversing rate bias (or heading bias) are factors that contribute to a biased random walk, it can be concluded that the microrobot exhibits a stronger tactic motion when a higher swimming speed is achieved.

## Discussion

We have studied the pH-tactic behavior of a large number of bacteria-propelled microrobots in a microfluidic channel with a stable spatial pH gradient. It has been demonstrated that the spatial pH gradient can effectively and consistently generate drift motion in the microrobotic system. To fully understand the biased motion of the microrobots and the mechanisms that produce the biased motion, we tracked individual microrobots and analyzed their trajectories. For free swimming bacteria, the tumble rate is biased with respect to swimming direction, yielding a heading bias, which in turn generates a pH-tactic or chemotactic drift velocity. However, unlike the clear run and tumble switching pattern of free swimming bacteria, the motion of bacteria-propelled microrobots can be described as an incessant translation with gradual changes in heading direction. From a trajectory analysis, the drift velocity of the microrobotic system under a stable pH gradient is found to result from two factors, namely, the heading bias and the swimming speed bias.

To explain how the heading bias is produced in the microrobotic system, we must understand the effect of pH on the flagellar tumbling rate. Free swimming bacteria increase their flagellar tumbling rates when moving towards unfavored pH regions or when sensing unfavored temporal pH changes. Since the assembly of bacteria onto micro polystyrene beads relies on physical adherence, we do not expect fundamental changes to the chemical sensing machinery of the bacteria after integration with the beads. Therefore, when the microrobot moves toward unfavored pH regions, the average flagellar tumbling rate of the attached bacteria tends to increase, and this introduces more disturbances to the motion of the microrobot by frequently changing the applied forces and torques. As a result, compared with motion towards favored pH regions, the microrobot maintains less consistency in its swimming direction when moving towards an unfavorable pH region; this leads to the reversing rate bias and hence the heading bias.

As we already discussed, the swimming speed bias reveals a bias in the propulsive force on the microrobot. Presumably, when the microrobot moves towards a favorable pH, less flagella are in a tumble state than when compared to moving towards an unfavorable pH. However, detailed observation of the flagella when the bacteria is attached to the microrobot is essential to fully understand the driving mechanisms. The dependencies of the motion bias on the swimming speed is potentially due to the fact that bacteria sense the temporal change of the ambient pH^34^; since the spatial pH profile is constant, it is the swimming velocity that determines the temporal pH gradient seen by the bacterial receptors. Therefore, the enhanced motion bias at higher swimming speeds could be explained by a stronger temporal pH gradient being sensed.

The work of Hu and Tu indicates that a common biochemical signaling pathway is responsible for different kinds of bacterial taxis behaviors, including pH-taxis, chemotaxis, and thermotaxis^25^. It is highly possible that appropriate chemical gradients and temperature gradients could also enable effective drift control of bacteria-propelled microrobotic systems with a similar design; the two methods should adhere to the same driving mechanisms that we have found in pH-taxis based control of biohybrid microrobots. To apply biohybrid microrobots in applications in bioengineering and medicine, reliable and efficient control of the microrobot at the swarm scale is a critical step. Our demonstrations of robust drift control using pH gradients expand the current scope of bacteria-propelled microrobotic control methods. The availability of a pH gradient or ease of deploying a gradient in the workspace will highly depend on the specific application. This work suggests the potential feasibility of applying pH-taxis, chemotaxis, and thermotaxis as control methods, and the appropriate method of control can be chosen based on the specific application.

## Methods

### Bacteria and growth conditions

*Serratia marcescens* (ATCC 274, American Type Culture Collection, Manassas, VA) was initially cultured to exponential growth phase in a nutrient broth (25 g Difco LB Miller Broth and 1 L deionized (DI) water, pH 7.0) on a shaker at 37 °C for 3.5–4 hours. Then an aliquot of 2.0 *μ*L of the liquid culture was transfered to an agar plate (25 g Difco LB Miller Broth, 6 g Bacto Agar, 5 g glucose, 1 L de-ionized water), followed by an incubation of the agar plate at 30 °C for 16–20 hours. After the culturing period, bacteria on the leading edge of the colony was either suspended in motility buffer for use in the bacterial pH-taxis study or suspended in a bead solution for the fabrication of the biohybrid microrobots.

### Microrobot fabrication

The microrobots were fabricated by randomly attaching bacteria to 3 *μ*m diameter fluorescent polystyrene beads (*ρ* = 1.05 g/cm^3^, Fisher Scientific, Inc.). To enable natural attachment between the bacteria and beads, the original coating of the beads was removed by alternately ultrasonicating the beads in deionized (DI) water or isopropyl alcohol (IPA, 50%) for a total of five cycles; residual IPA in the bead solution was removed by three more ultrasonication cycles with DI water. The washed beads were soaked in motility buffer at a volume concentration of 0.05%. The microrobots were assembled by placing an aliquot of 2.5 *μ*L bead solution onto the leading edge of the bacteria colony on the agar plate and gently pipetting 3–5 times to mix the bacteria and beads sufficiently. The solution was collected back immediately and incubated at room temperature for 5 minutes, allowing for random attachment of the bacteria to the beads. Then, the solution was diluted by adding 40 *μ*L of Percoll (*ρ* = 1.13 g/cm^3^, Sigma-Aldrich, St. Louis, MO) and 57.5 *μ*L of motility buffer to the solution. Percoll was added to increase the density of the fluid, thereby making the microrobots neutrally buoyant. The final solution was further diluted to achieve an appropriate concentration for vision tracking of the microrobots.

### Microfluidic setup fabrication

**Figure 7:**
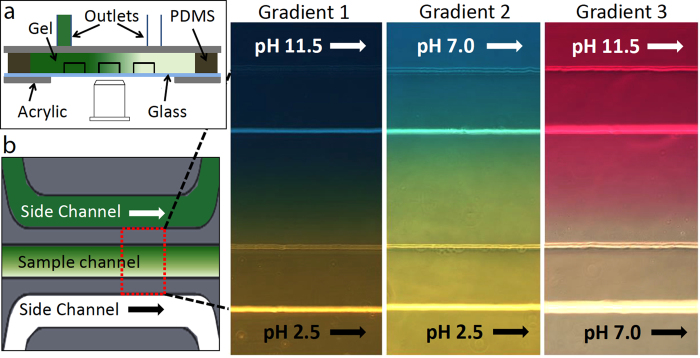
(**a**) Configuration of the three-channel diffusion-based pH gradient generator (side view). (**b**) Top view of the three parallel channels and the three gradient profiles visualized *in situ* by three appropriate pH indicators, where bright lines in the color profiles indicates the channel walls. The pH gradient in the sample channel was generated by pumping two fluids with different constant pH values into the side channels, while the sample channel was completely closed and quiescent. The pH indicators used to visualize the three gradients from left to right were Bromothymol Blue (sensitive pH 6.0–7.6, with color transitioning from yellow to blue), Bromocresol Green (sensitive pH 3.8–5.4, with color transitioning from yellow to blue) and Cresolphthalein (sensitive pH 8.2–9.8, with color transitioning from colorless to purple).

The microfluidic concentration gradient generator was assembled from a molded hydrogel chip containing the channel features^35^. To mold the hydrogel chip, a master mold of the channel patterns was fabricated by a standard soft lithography method. To increase the channel height, two layers of photoresist were used. The hydrogel chips were molded by pouring 4% (weight ratio) hot agarose (Eiken Chemical Co.) solution onto the silicon master mold, where the channel patterns were surrounded by polydimethylsiloxane (PDMS) enclosures. After the agarose gels were cured, the outlets of the source and sink channels were punched into the gel. Subsequently, the sample solution to be tested was carefully pipetted into the sample (middle) channel ensuring that there was neither overflow nor much vacant space left in the sample channel. The channel-patterned side of the agarose gel was covered with a cover slip immediately after loading the sample solution. To complete the assembly of the gradient generator, the agarose gel chip (including the diffusion section, a PDMS enclosure and a cover slip) was sandwiched between two acrylic panels as shown in Fig. 7(a).

### pH gradient generation and visualization

Unlike the generation of pH gradients by electrolysis^36^, a diffusion-based method can eliminate the electrical field induced effects on the motion of the microrobot. Therefore, a flow-free diffusion based gradient generator design^35^,^37^ was applied to fabricate the pH gradient generator. As shown in Fig. 7(a,b), the gradient generator consists of three parallel channels, namely the sample channel and two side channels. The width of the channels is 500 *μ*m and the height is around 200 *μ*m. The channels are separated from each other by two 250 *μ*m wide agarose gel ridges. A constant flow of fluid was pumped through the outlets of the two side channels, whereas the outlets of the sample channel were sealed to decrease undesired drift flows. A programmable syringe pump (Braintree Scientific Inc.) was used to pump fluid with different pH values into the two side channels at a flow rate of 5 *μ*L/min. Generation of a linear concentration gradient in the device has been fully calibrated and reported in our previous work^32^.

To create solutions with different pH values, either HCl or NaOH solution was added to motility buffer (0.01 M KH_2_PO_4_, 0.067 M NaCl, 10^−4^ M EDTA, pH = 7.0). Three stable pH gradients were generated by pumping motility buffer with different values of pH into the two side channels of our device. To verify the existence of a stable pH gradient in the sample channel, the same concentration of an appropriate pH indicator was added to all three channels. The pH gradients were visualized *in situ* and the pH transition in the sample channel was determined, as shown in Fig. 7(b). Based on the indicator color charts and the resulting color profiles, the following pH ranges were measured: Gradient 1 maintains a pH range between 6.0 (bottom) and 7.6 (top) in the sample channel, and a neutral pH region lies close to the center line of the sample channel; Gradient 2 maintains a pH range between 3.8 (bottom) and 5.4 (top), creating a more acidic environment along the bottom; the color profile of Gradient 3 indicates a pH range between 8.2 (bottom) and 9.8 (top), showing that the top is more alkaline. The pH gradients generated by the diffusion of ions were verified to be stable by the constant color profiles of the indicators after the estimated diffusion time. Since the time scale of ionization and recombination is negligible compared to that of diffusion, the diffusion time of H^+^ (0.9 min) and OH^−^ (1.6 min) across the two side channels was used to characterize the stabilization time of the pH gradients. In addition, we can treat the ionization of the sample solution to be quasistatic at each moment, i.e., there was no electric field induced by the diffusion of ions.

### Imaging and tracking

The samples were imaged using a 10x (fluorescence imaging of microrobots) or 40x (phase contrast imaging of bacteria) objective in an inverted microscope (Zeiss Axio Observer 100). Video data were captured at 88 fps (bacteria) and 5 fps (microrobots) by a digital camera (QICAM, QImaging) attached to the microscope. Both the captured bacteria and microrobots were located far away (≥10 body lengths) from any channel walls to eliminate wall effects on their motion. Two-dimensional (2D) motion of the microrobots was obtained from the video data using an in-house tracking program developed in MATLAB (R2012a, The MathWorks, Inc, Natick, MA). 2D bacterial swimming motion was tracked and analyzed with the methods described in our previous publications.^29^,^32^.

To calculate the COM-*y* of the microrobotic system for a given imaging frame, all the microrobots in the frame were detected by image processing and their 2D positions were determined. Then COM-*y* was calculated by averaging the *y* positions across all microrobots: 
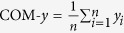
, where *n* is the total number of microrobots.

### pH-taxis drift velocity model

A particle’s movement along one direction, namely the *y*-axis, is modeled to determine the factors that can contribute to the drift velocity of a system exhibiting an inherent biased random walk. Assuming that the particle maintains different mean speeds when moving towards +*y* and −*y* directions, denoted by *v*_+*y*_ and *v*_−*y*_, respectively; the particle can switch its direction of motion in a way such that the portion of time it spends moving towards the +*y* direction, *t*_+*y*_, is different from the time it spends moving towards the −*y* direction, *t*_−*y*_. It is straightforward to describe the mean speed 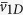 and the drift velocity *V*_*drift*_ (with +*y* be the default direction) of the particle.


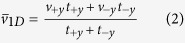



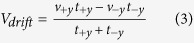


In Eq. (3), the drift velocity is essentially caused by the bias in swimming speed and the bias in the time spent in moving in a given direction. To include the two biasing factors, we define two coefficients: the coefficient of speed bias, *α* = *v*_−*y*_/*v*_+*y*_, and the coefficient of orientation bias, *β* = *t*_−*y*_/*t*_+*y*_. Substituting these coefficients into the two equations above, *V*_*drift*_ can be expressed in terms of the mean speed 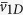,


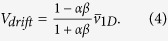


## Additional Information

**How to cite this article**: Zhuang, J. *et al*. pH-Taxis of Biohybrid Microsystems. *Sci. Rep*. **5**, 11403; doi: 10.1038/srep11403 (2015).

## Supplementary Material

Supplementary Information

Supplementary Video S1
