# Supplementary material for: pH-Taxis of Biohybrid Microsystems
Source: Sci Rep. 2015 Jun 15;5:11403. doi: 10.1038/srep11403 (PMC4466791; doi:10.1038/srep11403)
Supplement: Supplementary Information [file srep11403-s1.pdf]

pH-Taxis of Biohybrid Microsystems

Jiang Zhuang,[1] Rika Wright Carlsen,[1,2] and Metin Sitti[1,3,*]
[1] *Department of Mechanical Engineering, Carnegie Mellon University, Pittsburgh, PA 15213, USA*
[2] *Department of Engineering, Robert Morris University, Pittsburgh, PA 15108, USA*
[3] *Max Planck Institute for Intelligent Systems, Stuttgart 70569, Germany*

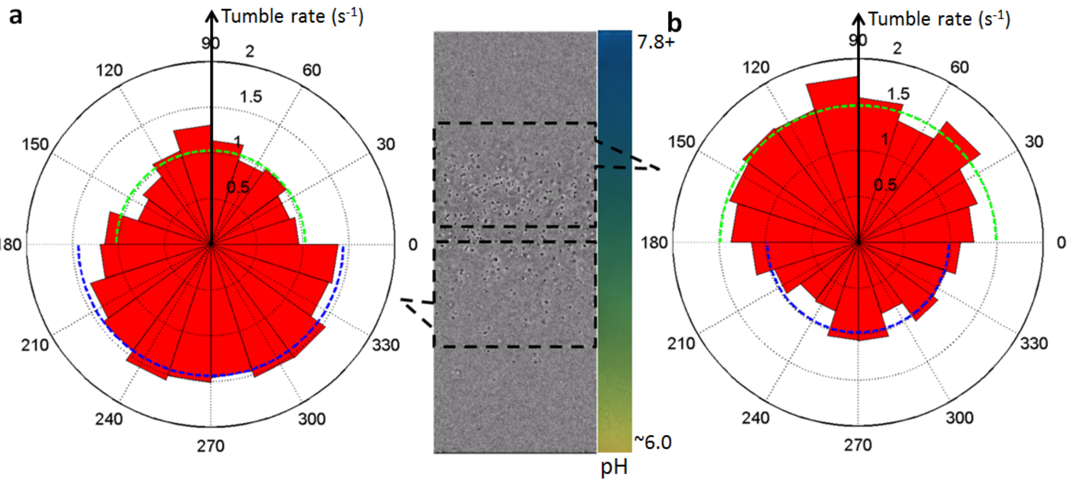

Fig. S1. **Bacterial tumble rate distribution (Gradient 1).** A phase contrast image of the bacterial distribution in the sample channel is shown in the middle; the image height corresponds to the full channel width. (a) Tumble rate distribution on the acidic side of optimal pH (∼7.0). (b) Tumble rate distribution on the basic side of optimal pH. In both cases, the average tumble rate when bacteria move towards the optimal pH is around 1.0 s^{-1}, while it is around 1.5 s^{-1} when moving towards unfavored pH regions.

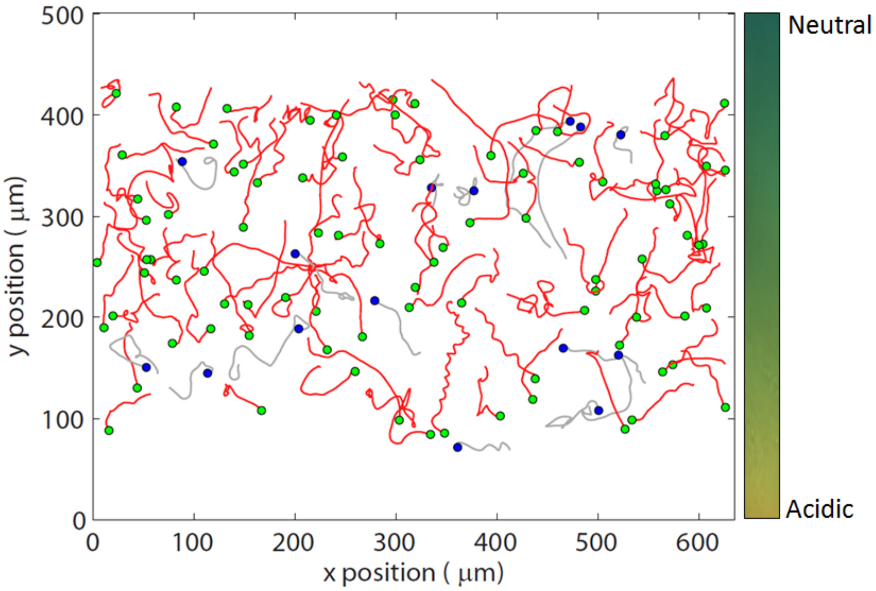

Fig. S2. **Sample swimming trajectories of microrobots.** The trajectories are randomly picked from microrobot samples undergoing unidirectional taxis away from an acidic pH condition (Gradient 2). The starting positions are marked by circles

* sitti@is.mpg.de (corresponding author)

(green and blue); the red trajectories have net y-displacements from acidic to neutral pH, while the gray ones have net y-displacements from neutral to acidic pH.

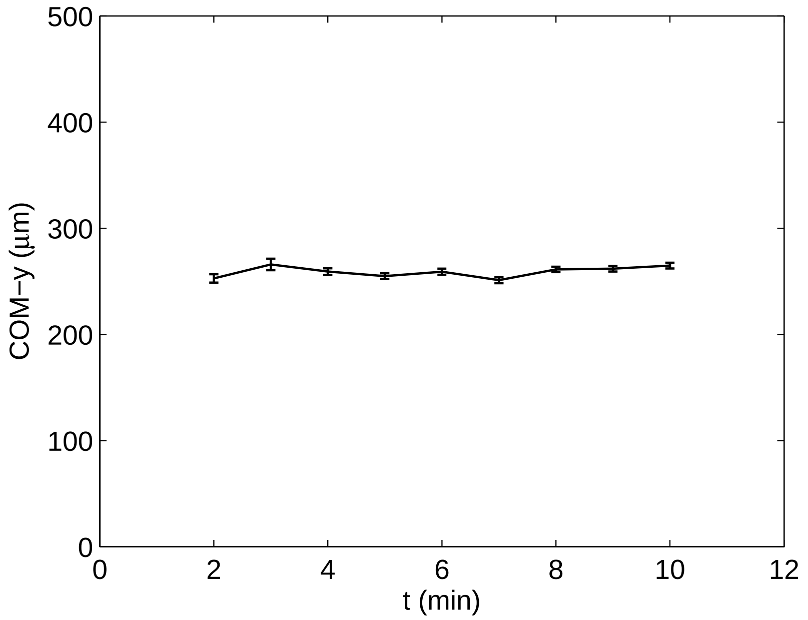

Fig. S3. **COM-y of microbeads without bacteria attached under a pH gradient (Gradient 1).** Without attaching bacteria, COM-y of the microbeads only fluctuates slightly around the mid-width of the channel, which reveals that there is no deterministic drifting for the bare microbeads under pH gradients. The error bar denotes the standard deviation of the COM-y of the captured frames within the corresponding one minute interval.
